# Porphyromonas gingivalis Fim-A genotype distribution among Colombians

**Published:** 2015-09-30

**Authors:** Sandra Moreno, Adriana Jaramillo, Beatriz Parra, Javier Enrique Botero, Adolfo Contreras

**Affiliations:** 1 School of Dentistry, University of Valle, Cali Colombia; 2 Periodontal Medicine Group, University of Valle. Cali, Colombia; 3 Department of Microbiology, University of Valle, Cali Colombia; 4 Faculty of Dentistry, University of Antioquia, Medellin Colombia

**Keywords:** Porphyromonas gingivalis, bacterial fimbriae, polymerase chain reaction, periodontitis, gingivitis

## Abstract

**Introduction:**

* Porphyromonas gingivalis* is associated with periodontitis and exhibit a wide array of virulence factors, including fimbriae which is encoded by the *FimA* gene representing six known genotypes.

**Objetive:**

To identify *FimA *genotypes *of P. gingivalis* in subjects from Cali-Colombia, including the co-infection with *Aggregatibacter actinomycetemcomitans, Treponema denticola*, and* Tannerella forsythia. *

**Methods:**

Subgingival samples were collected from 151 people exhibiting diverse periodontal condition. The occurrence of *P. gingivalis*, *FimA *genotypes and other bacteria was determined by PCR.

**Results:**

*P. gingivalis *was positive in 85 patients. Genotype *FimA II* was more prevalent without reach significant differences among study groups (54.3%), *FimA IV* was also prevalent in gingivitis (13.0%). A high correlation (*p*= 0.000) was found among *P. gingivalis, T. denticola, *and *T. forsythia *co-infection. The *FimA II *genotype correlated with concomitant detection of* T. denticola *and *T. forsythia*.

**Conclusions:**

* Porphyromonas gingivalis *was high even in the healthy group at the study population. A trend toward a greater frequency of *FimA II *genotype in patients with moderate and severe periodontitis was determined. The *FimA II *genotype was also associated with increased pocket depth, greater loss of attachment level, and patients co-infected with *T. denticola *and *T. forsythia*.

## Abstract (es)

**Introducción:**

*Porphyromonas gingivalis* es una bacteria asociada con la periodontitis. Expresa una amplia gama de factores de virulencia, incluyendo las fimbrias, las cuales están codificadas por el gen *FimA* que representa seis genotipos conocidos.

**Objetivo:**

Identificar los genotipos de *FimA* de *P. gingivalis* en pacientes de Cali - Colombia, incluyendo la co -infección con *Aggregatibacter actinomycetemcomitans*, *Treponema denticola *y* Tannerella forsythia* .

**Métodos:**

Se obtuvieron muestras subgingivales de 151 individuos con diferentes diagnósticos periodontales. La ocurrencia de *P. gingivalis,* los genotipos de *FimA* y otras bacterias se determinó por PCR.

**Resultados:**

*Porphyromonas gingivalis* fue positiva en 85 pacientes. El genotipo *FimA II* fue más prevalente, pero no hubo diferencias significativas entre los grupos de estudio (54.3%) , *FimA IV* fue el más frecuente en la gingivitis (13.0%). Una alta correlación (*p*= 0.000 ) se encontró entre *P. gingivalis , T. denticola y T. forsythia*. El genotipo *FimA II* estuvo correlacionado con la detección de *T. denticola *y* T. forsythia . *

**Conclusiones:**

*Porphyromonas gingivalis* tuvo una alta frecuencia incluso en el grupo de individuos sanos. Se encontró una tendencia hacia una mayor frecuencia de *FimA II* en pacientes con periodontitis moderada y severa. El genotipo *FimA II* también se asoció con una mayor profundidad de la bolsa, una mayor pérdida de nivel de inserción, y con los pacientes en los que se detectó co - infección con *T. denticola *y* T. forsythia*.

## Introduction

Periodontitis is an inflammatory disease that affects supporting tissue around teeth 1. One of the triggering factors of the disease is the persistence of the biofilm formed on the dental surfaces and the gingival margin 2.

In Colombia, according to the Third National Study on Oral Health in 1999, periodontal insertion loss is 50.2% and this generates increasing awareness to the health system because its high prevalence. 

*Porphyromonas gingivalis* is associated to the onset and progression of chronic and aggressive periodontitis 3-5, frequently present in patients with periodontal disease and is detected even in healthy subjects 6. *Porphyromona gingivalis* carry multiple virulence factors 4,7-8 and fimbriae is considered a key factor 7,9 composed by subunits of fimbrillin 10, located on the cell surface, which gives the bacteria the capacity to adhere to the supporting periodontal tissue, the acquired pellicle and to other bacterial species during biofilm formation and consolidation. The gene encoding the fimbriae is denominated* FimA* and six genotypes are known (I, Ib, II, III, IV, V) based on their nucleotide sequences 10.

In periodontitis subjects *FimA* *II *and* FimA IV *genotypes have been the more frequently identified in contrast to healthy subjects in whom the *FimA I *genotype is the most prevalent 11-14. 

This cross-sectional descriptive study seeks to determine the prevalence of *FimA* genotypes from *P. gingivalis* isolated from subgingival samples in health, gingivitis and chronic periodontitis subjects and determine its association to other periodontopathic microorganisms like A*ggregatibacter actinomycetemcomitans, Treponema denticola,* and* Tannerella forsythia*.

## Materials and Methods

Subgingival samples were taken from 151 subjects, selected by convenience. Sixty seven men and 84 women with age range 22-84 yrs old, (43.4 ±10.6 yrs), coming from 3 clinics: The Dental School at Valle University, The Dental Clinics of Santiago de Cali University, and one private dental clinic in Cali-Colombia. Subjects had different periodontal diagnoses (25 healthy, 77 gingivitis and 49 chronic periodontitis). The patients authorized participation. The study was approved an IRB committee at the Faculty of Health -Valle University-, protocol No. 012-010. Selection was made of subjects who had more than 15 teeth, who had not taken antibiotics, corticoids, and/or AINES at least three months prior to be included in the study, without systemic diseases and who had not received periodontal treatment and/or professional dental cleaning at least six months prior to participating in the study. 

### Periodontal examination and sampling

A full-mouth periodontal probing chart was performed by two calibrated periodontist accordingly to the diagnostic criteria of the AAP 1999. The measurements of probing pocket depth and clinical attachment loss was recorded at six points around each tooth. The variables considered for clinical diagnosis were probing depth, clinical attachment loss, the rate of bleeding and the number of teeth. Healthy subjects were considered had not signs of inflammation, probing depth sites equal or less than 3 mm, no bleeding on probing without clinical attachment loss. Gingivitis patients presented clinical signs of gingival inflammation and edema, bleeding on probing without clinical attachment loss. Periodontitis patients presented swelling, bleeding on probing and probing depth of 4 mm in at least one tooth (localized) or more than 50% of the teeth (generalized). After removal of the supragingival plaque with sterile gauzes, two sterile paper tips were introduced in the deepest pockets (>4 mm) in patients with periodontitis and in the mesial vestibular surface of the first four molars in healthy subjects and patients with gingivitis, leaving them in the sulcus for one minute. Thereafter, the paper tips were deposited in Eppendorf tubes and cryopreserved at -20° C until processing 14,17

### DNA extraction and PCR

Bacterial DNA extraction from the paper tips was performed through adsorption to silica particles, according to Boom's 1989 protocol, and the *P. gingivalis* genotyping procedure was carried out via the Polymerase Chain Reaction (PCR) technique, using published reports 16-18. First, DNA extraction was confirmed by using specific generic primers for 16S rRNA; then, the presence of DNA from *P. gingivalis* was confirmed with specific primers 16-18 and, thereafter PCR was performed with the specific primers for each *FimA I, II, III, IV, V, *and *Ib *genotype*, *as widely reported in literature 11,14-17. 

DNA from *P. gingivalis* ATCC33227 (*FimA* I), W83 (*FimA IV*), ATCC33279 (*FimA* Ib) strains was used as positive controls 14. For positive controls of *FimA* *II *and* FimA III* genotypes, two clinical isolates were used numbered 486 and 723, which were typified and donated by the Microbiology laboratory at Bosque University 13.

The PCR tests to study the *FimA *genotype were conducted in a thermocycler (AXYGEN) with the following amplification program: initial denaturing at 95° C for 5 min, followed by 36 cycles at 94° C for 30 s, 60° C for 30 s, and 72° C for 30 s, and a final extension at 72° C for 7 min. Besides the *FimA* genotypes from *P. gingivalis,* the same samples were also used to identify the presence of other periodontopathic bacteria, like *A. actinomycetemcomitans,* *T. denticola *and* T. forsythia* through a single round PCR technique using specific primers for the 16S rRNA gene from each of the three microorganisms before mentioned. The PCR products were separated electrophoretically in agarose gels and the DNA bands were stained with SYBR safe and visualized via UV light in a trans-illuminator (Invitrogen). The results were documented through photographic registration. Identification of *FimA* genotypes was carried out according to the molecular size of the amplification bands obtained, compared to the respective positive control 14. 

### Standardization and analytic sensitivity for the PCR for P. gingivalis Fim A gene detection 

To determine the limit of detection of the *FimA* gene from *P. gingivalis *by PCR technique, 

a serial and tittered dilutions of known bacterial genotypes coming from clinical isolates of *FimA II *and *FimA III, *or control strains ATCC33279 (*FimA Ib*), W83 (*FimA IV*), and ATCC 33227 (*FimA I*) were done. Likewise, PCR sensitivity for *FimA* genotypes in clinical samples was determined by experimental inoculation of GCFs negative for *P. gingivalis* using diverse dilutions of prototype strains in spike samples. 

### PCR specificity for the P. gingivalis FimA gene

Primers and PCR specificity of *FimA* genotypes (*I, II, II, IV, *and* Ib)* was probed against other periodontopathic bacteria like *A. actinomycetemcomitans *(D11-s1)*, T. denticola (ATCC. 43056), *and* T. forsythia *(ATCC 43037).

### Statistical analyses

Pearson´s Chi squared statistical test was used to compare the frequencies of the *FimA* genotypes. To analyze the frequency of other periodontopathogens, Fisher's exact test was applied taking in account that sample size was below 20. Clinical attachment loss or CAL was analyzed by Student's T test and quantitative variables like probing pocket depth - PPD, and Bleeding on Probing - BOP, number of teeth, and age were analyzed by using Wilcoxon's test and Mann-Whitney test based on previous testing of Normal distribution.

## Results 

### PCR sensitivity and specificity for the P. gingivalis FimA gene

The detection limit of the *FimA* gene was determined to detect up to 50 bacterial cells in the dilutions carried out from a pure culture from each *P. gingivalis *strain available. However, the detection limit increases up to 500 cells when the bacterial dilutions were inoculated in GCF possibly by the action of PCR inhibitors or DNAases. High specificity was found between the primers used and the representative strains of each of the *FimA* genotypes studied.

###  Frequency of P. gingivalis

A total of 85 subjects were positive for *P. gingivalis *(56.3%) being the bacteria more prevalent in women 57.6% of that in men 42.3%. Frequency of *P. gingivalis* by diagnoses was 52.0% for healthy subjects, 59.7% for gingivitis patients and 53.1% for chronic periodontitis (Table 1).

**Table 1.** Prevalence and Distribution of *FimA* genotypes of *P. gingivalis* according to periodontal diagnosis (AAP 1999).

Genotypes	Periodontal Diagnosis						
	Healthy		Gingivitis		Periodontitis		*p* value*
	**n	(%)	n	(%)	n	(%)	>0.050
*P. gingivalis*	13	52.0	46	59.7	26	53.1	0.681
*FimA I*	2	15.4	12	26.1	3	11.5	0.301
*FimA II*	6	46.2	25	54.3	15	57.7	0.792
*FimA III*	2	15.4	2	4.3	2	7.7	0.386
*FimA IV*	1	7.7	6	13.0	0	0.0	0.154
*FimA Ib*	3	12.0	15	19.5	2	4.1	0.057
*FimA V*	0	0.0	0	0.0	0	0.0	
Not tipificable	5	38.0	10	21.7	8	30.7	0.428
fimA I, II, Ib	1	7.7	8	17.4	0	0.0	0.066
fimA III, Iv, Ib	1	7.7	0	0.0	0	0.0	0.079
fim A I, III, Ib	1	7.7	0	0.0	0	0.0	0.079
fimA II, IV	0	0.0	2	4.3	0	0.0	0.377
fimA I, II	0	0.0	1	2.1	2	4.3	0.406
fim A III, Ib	0	0.0	0	0.0	1	2.1	0.350
fim A III, IV	0	0.0	1	2.1	0	0.0	0.616
fim A I, Ib	0	0.0	4	8.7	1	3.8	0.375

* Pearson´s Chi squared statistical test.

**The values of n not add up to total shown since the same subject can express various genotypes time.

###  Frequency and distribution of P. gingivalis FimA genotypes 

**Figure 1.**
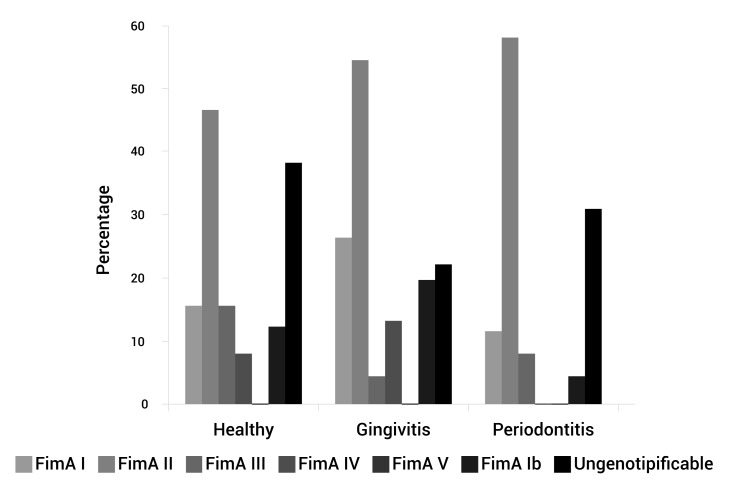
Genotypes *FimA* *P. gingivalis* frequency according periodontal diagnosis.

The most frequent genotype was *FimA II *(54.3%), followed by *FimA Ib *(23.5%), *FimA I *(20%), *FimA IV* (8.2%), and *FimA III* (7.1%). No positive samples were found for the *FimA* *V* genotype. To the 85 patients positive for *P. gingivalis*, 23 (27%) were negative for the six genotypes studied hence, assigned as the unknown genotype or non/tipificable (Table 1). In the three groups: healthy, gingivitis, and periodontitis patients, the percentages of fimA II genotypes were 46.2%, 54.3%, and 57.7%, respectively (Table 1, Fig. 1). 

 The *FimA II* genotype was more frequent as increasing the severity of periodontitis (AAP 1999), although no statistically significant difference was reached (*p*= 0.813). We also analyzed the prevalence and distribution of the different *FimA* genotypes according to the clinical variables analyzed and it was found that *FimA* *II *and* FimA III *genotypes were linked to a high PD per site. The *FimA II* was related to a higher CAL and tooth loss. *FimA III* genotype was related to higher BOP (Table 2). 

**Table 2.** Relation between *FimA* genotypes and clinical variables.

Genotypes	Clinical variables*			
	PD	CAL	BOP	NoTe
*FimA I*	4.3	4.2	55.8	25.1
*FimA II*	4.6	4.5	53.4	23.2
*FimA III*	4.6	4.0	63.5	24.0
*FimA IV*	4.4	4.3	61.8	23.4
*FimA Ib*	4.2	4.0	56.0	24.0

*All data are mean

PD: probing pocket depth site (mm)

CAL: clinical attachment loss site (mm)

BOP Bleeding on Probing (%)

NoTe: number of teeth

Eleven patients were positive for three *FimA* genotypes and 12 patients harbored two fimA genotypes, meaning that coinfection is a not rare event (Table 1).

###  Prevalence of other periodontopathogens

It was a high prevalence of *T. forsythia* (65.3%) in the periodontitis patients while its prevalence in healthy patients and gingivitis patients was 56.0% and 68.8% respectively. The prevalence of *T. denticola* in healthy, gingivitis and periodontitis patients was 40.0%, 58.4% and 40.8% respectively and for *A. actinomycetencomitans* was 8.0% in healthy patients, 20.8% in gingivitis and 14.2% in periodontitis (Table 3). 

**Table 3.** Positive co-infection with other periodontopathogens and *FimA* genotypes.

		Species*		
		A. actinomyc**	T. denticola	T. forsythia
Periodontal diagnosis	Healthy	8.0	40.0	56.0
	Gingivitis	20.8	58.4	68.8
	Periodontitis	14.2	40.8	65.3
	*P .gingivalis*	64.0	74.7	72.7
Genotypes FimA			
	*FimA I*	31.2	21.4	22.2
	*FimA II*	68.8	55.3	52.8
	*FimA III*	6.7	7.1	5.5
	*FimA IV*	6.7	8.9	6.94

*Percentage

**A. actinomycetemcomitans

###  Co-infection of P. gingivalis and FimA genotypes with A. actinomycetemcomitans, T. denticola, and T. forsythia 

A high correlation was observed between *P. gingivalis, T. denticola, *and* T. forsythia*, (*p*= 0.000). The co-infection of these microorganisms was observed in higher percentages for the *FimA II* genotype with prevalence in the samples positive for *A. actinomycetemcomitans* (68.7%), *T. denticola *(55.3%), and *T. forsythia* (52.8%), although this relationship was not statistically significant (Table 3). 

##  Discussion

### Frequency of P. gingivalis

 The results of this study in the* P. gingivalis* frequency differ from those reported by Amano *et al.,* 14,15, Guo *et al.,* 19, Wu *et al.,* 22, and, Zhao *et al.,* 23, but are similar to those reported by Beikler 20 and Missailidis *et al* 16. 

High *P. gingivalis* prevalence in healthy patients is common in the Colombian population, according to that reported by Botero *et al.,* 24, Lafaurie *et al., *25, and Mayorga *et al.,* 26. This finding could be influenced by the ethnicity, low sanitation and poverty. Most of the patients in our study live on wages below $400- $500 US dollars per month. This hinders access to dental services, worsening periodontitis, causing delay or lack on dental treatment and favoring perhaps horizontal and vertical transmission of important periodontal pathogens. 

### Frequency and distribution of P. gingivalis FimA genotypes

In general, the most frequent genotype was *FimA II*, which was detected in 46 samples (54.1%). 

Healthy patients revealed prevalence for *FimA II* followed by *FimA I* These results largely differ from studies published in literature, that reported lower frequencies of *P. gingivalis* in healthy patients 16,20,21, as well as low frequencies of the *FimA II* genotype and high frequencies of the *FimA* *I, FimA III, *and* FimA V* genotypes 14,16,22,23, considering these genotypes as low pathogens (Table 1, Fig. 1). 

 It is also worth considering the importance of conducting studies on genotype expression through, for example, Real Time-PCR and to determine the relationship between the phenotype and the genotype. Another important issue to bear in mind is that possibly the most virulent genotypes are present during initial stages of the periodontal disease, where the process of colonization and infection begins, as well as the stimulus of the immune and inflammatory response, which over time will generate damage and destruction of tissue. 

The difference between the gingivitis and healthy groups may be associated to the bacterial load or the combination of virulence factor of diverse organisms. It might be either important to do a follow up of the populations to unveil if genotypes remain constant or varies over the time and after treatment. Further studies are required to prove these hypotheses.

Results in the gingivitis and periodontitis groups with respect to higher prevalence (*FimA II* genotype), specifically in generalized gingivitis moderate periodontitis, and severe periodontitis coincide with studies from Japan 15,16,18, China 19,23, Germany 20, Norway, the US, Canada, and The Netherlands 21, Brazil 11,16, Mexico 17, and, Colombia 13. The second most common genotype in these groups (*FimA I*), which in different studies has been associated to healthy individuals, revealed values of 26.1 and 11.5%, respectively, (Table 1) which is quite similar to that reported by Missailidis *et al.,* 16 and Davila *et al.,* 17, who reported this genotype as the second most prevalent in patients with gingivitis and periodontitis.

In our study there were no statistically significant differences in the distribution of genotypes for periodontal diagnosis. However a main reason could be the sample size. Moon *et al*., reported the finding that primers specific for *fimA II* could cross react with *FimA Ib* genotype leading to errors 27. The same research group reported the *FimA II* prevalence in Koreans reporting 44.5% in healthy subjects and 67.3% in periodontitis and concluding that results of previous studies should be reviewed as entailed to an overestimation of the relative risk for *FimA II* genotype. The authors suggest that healthy subjects carry genotype II have increased risk of developing periodontal disease 28. Interestingly, these results are very similar to our findings.

All the positive samples for *P. gingivalis* were negative for the *FimA V *genotype (Table 1). This result coincides with that reported by Missailidis *et al.*, 16 and by Enersen *et al.*, 29, but differs from the results from Japan 18, and from China 23 who reported prevalence of 16 to 29%; however, very low prevalence of this genotype has been reported (1.0-3.9%) in studies published in Germany 20 and Norway 21. 

It is worth highlighting that in developing this study *P. gingivalis* *FimA V* control strain was not available and although PCR was performed using specific primers for this genotype, no sample turned out positive. The lack of the *FimA V* strain for the positive control is a limitation in our study. 

Upon comparing the clinical variables, it was found that the *FimA III* genotype was most related to increasing PD, increasing CAL and bleeding on probing. Nonetheless, when comparing the relationship of the genotypes with the probing depth and insertion level loss at sampling sites (Table 2), it was found that the *FimA II* genotype was related to greater PD and greater CAL being similar to previous reports 11,14,29.

The presence of more than one genotype has been widely reported in literature 16,30. The current study presented 23 samples, mostly distributed in the gingivitis group with frequencies at 9.4% of the total for two genotypes and 9.4% for three genotypes (Table 1). This heterogeneous distribution of *P. gingivalis* genotypes on the population could be associated to ethnic differences. 

###  Prevalence of periodontopathogens: A. actinomycetemcomitans, T. denticola, and T. forsythia 

This study found high prevalence of the three microorganisms in the whole sample*, *being most prevalent in the gingivitis and periodontitis group as compared to healthy individuals, which agrees with other studies in Colombia done by Botero *et al., *24 and Lafaurie *et al* 25.

###  Co-infection of P. gingivalis genotypes with T. denticola, T. forsythia, and A. actinomycetemcomitans 

The current study found positive association among the three microorganisms from the red complex described Socransky *et al.,* 31 and corroborated by others 23-25 (Table 3). The study also found a high percentage of positive samples for *P. gingivalis *and *A. actinomycetemcomitans*; these two microorganisms have been considered important etiological factors during the onset and progression of periodontal disease 32. Of the samples positive for *P. gingivalis* and the other three microorganisms studied, the majority were from the *FimA II* genotype, which could indicate a higher co-infection ratio of this genotype with *T. denticola, T. forsythia*, and* A. actinomycetemcomitans. *These results are similar to those obtained by Zhao *et al.,* 23. 

## Conclusions 

*FimA II *of *P. gingivalis* was detected in gingivitis, periodontitis and healthy subjects, and was also associated with periodontal disease severity. *Porphyromonas gingivalis* was associated with *T. denticola *and* T. forsythia *and *FimA II* genotype was frequent in patients that presented co-infection with these periodontal pathogens*.*
